# What Is Going on in Indoor Air Quality of a University Hospital in Northern Cyprus?

**DOI:** 10.4314/ejhs.v33i3.18

**Published:** 2023-05

**Authors:** Meryem Güvenir, Ufuk Kaya, Dilek Özçelik, Kaya Süer

**Affiliations:** 1 Faculty of Health Sciences, Cyprus Health and Social Sciences University, Morphou, Cyprus; 2 Department of Nursing, Faculty of Health Sciences, Cyprus Health and Social Sciences University, Morphou, Cyprus; 3 Department of Nursing, Faculty of Health Sciences, Cyprus Health and Social Sciences University, Morphou, Cyprus; 4 Department of Infectious Diseases and Clinical Microbiology, Faculty of Medicine, Near East University, Nicosia, Cyprus

**Keywords:** Indoor air quality, hospital, bacteria

## Abstract

**Background:**

There are two main purposes of microbial monitoring of the inanimate hospital environment. One of them is to monitor hygiene standards, and the second is to determine the presence of nosocomial pathogens. This study was aimed to investigate the indoor environment of the different departments of a university hospital.

**Methods:**

The prospective study was conducted in a university hospital, a teaching hospital with 143 beds and 30 ICU beds, and a hospital with 6 different wards in Northern Cyprus. Active air sampling was done by using an air IDEAL 3P device. Air samples were taken at 38 points defined before in different parts of the hospital.

**Results:**

Our results showed a low level of microorganisms' numbers in microbial airborne communities in a university hospital. Moreover, our results indicated that the temperature of the hospital's indoor environment is not significantly related to the airborne microbial community. On the other hand, our results indicate that the Aspergillus species were mostly isolated in the drug preparation room.

**Conclusion:**

The studies have shown that one of the causes of hospital infections is the microorganisms entering the airborne microbial communities. In this case, epidemiology and pathogenesis of airborne microbial communities should be understood.

## Introduction

It is a certain fact that microorganisms adversely affect air quality, ecosystem, and human health. Especially, the indoor environment is the cause of the spread of microbial agents. These microbial agents are transmitted by droplets and/or by air. These microorganisms that can be suspended in the air can be bacteria, viruses, fungi, toxins, and plant remains (1, 2). This microflora in hospital rooms causes a serious risk of contamination (3).

Nowadays, hospital infection is still a major health problem. Microorganisms, especially in the air, are a great danger for nosocomial infections. This does not cause a thread only for patients, but may be for the relatives and patient visitors (3, 4). The low air quality stands out as a big threat to human health (5).

Bacteria in the air are the major factors in the occurrence of nosocomial infections (4). Air quality is affected by multiple factors that include ambient air, location, hygienic rules, activities of humans, soil, microclimatic factors, and ventilation (6). Air quality also affects many factors. These factors are listed as follows: pollutant pathways, heating, ventilation and air conditioning (HVAC) system, air quality, building construction and construction materials, temperature, contaminant sources, occupants, and pollutant pathways (7).

In the study of Osman et al. (2017), it was stated that *Aspergillus* and *Penicillium* are common fungal species in the air in powder form, and they constitute important sources of bacteria and fungi in hospital air (2). In the study of Okten and Asan (2012), *Cladosporium, Alternaria* and *Penicillium,* colonies were found to be the most common fungal genera (3). In this study, the aim is to investigate the indoor environment of the University Hospital. For this, data collection made in some different departments. These departments are inpatient services, laboratories, outpatient services, operating rooms, medicine preparation rooms, emergency room, intensive care units.

## Materials and Methods

**Setting and study design/hospital**: This prospective study was conducted in a university hospital, a teaching hospital with 143 beds and 30 Intensive Care Units (ICU) beds, and a hospital with six different wards in Northern Cyprus. All ICUs of the facility were being ventilated by a high-efficiency particulate arresting air conditioning system. Human beings were not included as research subjects in this study. The hospital where the research was conducted is a university training and research hospital. The university hospital, which has technologically advanced facilities, has many departments such as emergency service, various intensive care units, 10 operating rooms, various inpatient services, polyclinics.

**Air sampling**: Active air sampling was done by using an air IDEAL 3P device (BioMérieux, Marcy-l'Étoile, France) according to the manufacturer's instructions. This device is an impactor-type instrument that indoor air is aspirated through a grid perforated with a pattern of 286 calibrated holes. The airstreams containing microbial particles are directed onto the surface of a 100-mm agar plate. Use of the air IDEAL 3P device was approved by a third-party institution to satisfy International Organization for Standardization 14698-1 requirements for the control of clean room environment; it was shown that 100% of particles above 5 µm were efficiently collected by using the reference air sampling method of the UK Health Protection Agency (12).

Air samples were taken at 38 points defined before in different parts of the hospital. Moreover, before the samples were taken, the degree of humidity and the temperature were measured and reported for each room. Care was taken to take the air sample from the same specific room in each section. Thessare drug preparation rooms, a patient room, waiting area for the patient and/or patient relatives in the department, some polyclinics and consumable warehouses. For this, units such as inpatient services, emergency room, operating rooms and intensive care units were used.

**Identification of the microorganism**: Agar plates were incubated for 48 h at 37 °C; Columbia blood agar (OXOID) with 5% blood was used for all samples. The air sampler was cleaned with 82% ethanol and 0.5% chlorhexidine swabs before and after taking the next samples. Agar plates were left overnight in the air-sampler and thereafter incubated at 37 °C for a minimum of 24 h to control for contamination. Next, any growing microorganisms were counted, and colony-forming units were calculated per meter^3^ air. Also, colony numbers were calculated based on the manufacturer's instructions to determine the most probable number of microorganisms collected (Statistical correction of the CFU value). The European Commission Report defined the limits for bio-aerosols as follows: 0 undetectable, 1-499 CFU/m^3^ low, 500-999 CFU/m^3^ medium and > 1000 CFU/m^3^ high (13).

**Statistical Analysis**: The SPSS (ver. 18, IBM) software was used for the statistical analysis. Frequency and average analysis tests were used to determine the number of colonies, mean ± standard deviation, median, minimum and maximum values of humidity and temperature parameters. The Pearson Chi-square test was used to find the difference between the number of colonies, humidity, temperature, and fungi (p < 0.001, p < 0.05).

**Ethical statement**: Ethics committee approval of the study was obtained from the Scientific Research Ethics Committee of Cyprus Health and Social Sciences University (KSTU//2022/92).

## Results

A total of 38 air samples were collected during the study period, of which 4 were from the ICUs and 34 were from different departments of the hospital, such as the patient room, dialysis room, storage room, and emergency room. The values of the number of bacteria and fungi (CFU/m3) were measured together with the microclimatic parameters such as temperature and humidity. The colony number of the bacteria is shown in Table 1. The European Commission Report defined the limits for bio-aerosols as follows: 0 undetectable, 1-499 CFU/m^3^ low, 500-999 CFU/m^3^ medium and > 1000 CFU/m^3^ high (13). In our study, the concentrations of bacteria varied and ranged from 1 CFU/m3 to 50 CFU/m^3^. The average level of bacteria was 22.76 CFU/m3. These figures, which emerged in line with the findings obtained from the study, are described as ‘low’.

**Table 1:** Statistical summary of bacterial and fungal counts and microclimatic parameters

	Colony Number of the bacteria (cfu/ml)	Most Probable Number of microorganisms collected. Statistical correction of the CFU value	Humidity (%)	Temperature (°C)
Mean	22.76	24.53	34.63	24.73
SD	18.40	20.46	1.21	0.98
Median	15.0	15.41	35.0	24.6
Minimum	1	1	32	22.90
Maximum	50	55.29	37	26.30

As a result of the study, it was determined that *Aspergillus flavus* was present in 16/38 of the services and departments where air analysis was made. These services and departments include gynecology service medicine preparation room, gynecology service patient room, gynecology service equipment/storage room, cardiology service medicine preparation room, surgery service medicine preparation room and equipment/storage, internal medicine service medicine preparation and equipment/storage room, geriatrics service medicine preparation room, emergency room's equipment/storage room, drug preparation room, bronchoscopy operation room, endoscopy operation room, gastroenterology polyclinic, patient operation room and disinfection room.

Correlation results of the Colony Number of the bacteria (cfu/ml), Most Probable Number of microorganisms collected, fungi, and humidity are presented in Table 2. In this table there is a positive correlation between statistical correction of the CFU value (r = 1.000). At the same time, statistical significance was obtained (p = 0.000). A positive correlation was determined between Colony Number of the bacteria (cfu/ml) and Fungi (r = 0.540). At the same time, statistical significance was found (p = 0.000). There is a positive correlation between the CFU value and Fungi (r = 0.541), and statistical significance was found between them (p = 0.000). A negative correlation was determined between Humidity (%) and Temperature (°C) (r = -0.581), and statistical significance was determined between them (p = 0.000).

**Table 2:** Correlation results of the Colony Number of the bacteria (cfu/ml), Most Probable Number of microorganisms collected, fungi and humidity

	Correlation	Colony Number of the bacteria (cfu/ml)	Most Probable Number of microorganisms collected. Statistical correction of the CFU value	Fungi	Humidity (%)	Temperature (°C)
**Colony Number of** **the bacteria (cfu/mL)**	**r**	-				
	**p**	-				
**Most Probable** **Number of** **microorganisms** **collected. Statistical** **correction of the CFU** **value**	**r**	**1.000** **	-			
	**p**	**0.000**	-			
**Fungi**	**r**	**0.540** **	**0.541** **	-		
	**p**	**0.000**	**0.000**	-		
**Humidity (%)**	**r**	0.144	0.147	0.197	-	
	**p**	0.387	0.377	0.235	-	
**Temperature (°C)**	**r**	0.064	0.063	- 0.059	**-0,581** **	-
	**p**	0.704	0.708	0.725	**0.000**	-

* p<0.05

** p<0.01

## Discussion

Bioaerosols are defined as natural particles of artificial or biological origin suspended in the air. Viable particles are transported by air form of a single cell as well as in a cluster of 1-10 µm microorganisms (8). There are different types of biological materials in the indoor environment such as fungus and bacteria (9). Therefore, indoor air quality is more important for the hospitals and other healthcare facilities than buildings (10). Microbiological monitoring in the indoor environment of hospitals can be used to evaluate the efficacy of routine cleaning and disinfection processes (11). Globally, the number of studies is limited to evaluate the microbial quality of indoor air in a hospital (or hospital environment). However, Martins-Diniz et. al. indicated that the purpose of the air monitoring study to be carried out in hospitals is to determine the source of pollution and detecting potential microorganism agents that can be found in hospital air (12). The aim of this study was to investigate indoor environments of the different departments and ICUs of the university hospital.

Environmental factors such as temperature and humidity have important effects on the microbial community (13). Gao XL et al. reported that environmental factors were significantly correlated with airborne microbial community composition (14). In addition, R.M. Bowers et.al (15) and S.W. Kembel et.al (16) pointed out that temperature and relative humidity are closely related to the airborne microbial. In the current study, the results of the correlation analysis of the relationship between the concentration of bacteria and relative humidity, are given at the level of significance (p = 0.377). According to Pearson's correlation coefficient, there is a positive correlation (r = 0.147) between the concentrations of bacteria and relative humidity. However, the results of this study indicate that there is no significant correlation between temperature and airborne microbial communities.

Fungal spores and *Aspergillus* species, which can be found in unfiltered air, contaminated dust, food, and pots in patient rooms, can be very common in hospital environments (17, 18). In our study, the results of correlation analysis between fungi and relative humidity are given with the level of significance (p = 0.000). There is also a positive correlation (r = 0.541) between the concentrations of fungi and relative humidity. In a study conducted in Greece, air, surface, and tap water samples were performed in the four departments (hematology, pediatric oncology, and pediatric intensive care unit) in which high risk patients were stayed. After air sampling, *Aspergillus niger, Aspergillus flavus* and *Aspergillus fumigatus* were found to be 25.9%, 17.7% and 12.4%, respectively (19,). Interestingly, our results indicate that *Aspergillus* species were mostly isolated in the medicine preparation room. Our observation was that this medicine preparation room is small and has no open window. Therefore, it will be a suitable characteristic for growing the fungi such as *Aspergillus spp*. For this reason, these rooms should be disinfected more than other departments (20).

In recent years, nosocomial infections in hospitals have increased and have caused significant morbidity and mortality. The studies have shown that one of the causes of hospital infections is the microorganisms entering the airborne microbial communities. With new and more comprehensive studies to be done, understandings of the epidemiology and pathogenesis of airborne microbial agents can be provided. This study is limited to the air samples taken from the units in the designated department of a private hospital in Northern Cyprus.
